# Regional perspectives on the coordination and delivery of paediatric end-of-life care in the UK: a qualitative study

**DOI:** 10.1186/s12904-023-01238-w

**Published:** 2023-08-16

**Authors:** Andrew Papworth, Julia Hackett, Bryony Beresford, Fliss Murtagh, Helen Weatherly, Sebastian Hinde, Andre Bedendo, Gabriella Walker, Jane Noyes, Sam Oddie, Chakrapani Vasudevan, Richard G. Feltbower, Bob Phillips, Richard Hain, Gayathri Subramanian, Andrew Haynes, Lorna K. Fraser

**Affiliations:** 1 Department of Health Sciences, Martin House Research Centre, University of York, Heslington, YO10 5DD York, UK; 2 Department of Health Sciences, University of York, York, YO10 5DD UK; 3 Social Policy Research Unit, University of York, York, YO10 5DD UK; 4 Hull York Medical School, University of Hull, Hull, HU6 7RX UK; 5 Centre for Health Economics, University of York, York, YO10 5DD UK; 6 Parent and Public Involvement member, York, UK; 7 School of Medical and Health Sciences, Bangor University, Bangor, LL57 2EF UK; 8 Bradford Teaching Hospitals NHS Foundation Trust, Bradford, BD9 6RJ UK; 9 Leeds Institute for Data Analytics, School of Medicine, University of Leeds, Leeds, LS2 9NL UK; 10 Centre for Reviews and Dissemination, University of York, York, YO10 5DD UK; 11 All-Wales Paediatric Palliative Care Network, Cardiff and Vale University Health Board, Cardiff, CF14 4XW UK; 12 College of Human and Health Sciences, Swansea University, Swansea, SA2 8PP UK; 13 Manchester University NHS Foundation Trust, Manchester, M13 9WL UK; 14 Cicely Saunders Institute, Kings College London, Bessemer Road, London, SE5 9PJ, UK

**Keywords:** End of life care, Paediatrics, Palliative care, Child health services, Qualitative

## Abstract

**Background:**

Provision of and access to paediatric end-of-life care is inequitable, but previous research on this area has focused on perspectives of health professionals in specific settings or children with specific conditions.

This qualitative study aimed to explore regional perspectives of the successes, and challenges to the equitable coordination and delivery of end-of-life care for children in the UK.

The study provides an overarching perspective on the challenges of delivering and coordinating end-of-life care for children in the UK, and the impact of these on health professionals and organisations. Previous research has not highlighted the successes in the sector, such as the formal and informal coordination of care between different services and sectors.

**Methods:**

Semi-structured interviews with Chairs of the regional Palliative Care Networks across the UK. Chairs or co-Chairs (*n* = 19) of 15/16 Networks were interviewed between October-December 2021. Data were analysed using thematic analysis.

**Results:**

Three main themes were identified: one standalone theme (“Communication during end-of-life care”); and two overarching themes (“Getting end-of-life services and staff in the right place”, with two themes: “Access to, and staffing of end-of-life care” and “Inconsistent and insufficient funding for end-of-life care services”; and “Linking up healthcare provision”, with three sub-themes: “Coordination successes”, “Role of the networks”, and “Coordination challenges”). Good end-of-life care was facilitated through collaborative and network approaches to service provision, and effective communication with families. The implementation of 24/7 advice lines and the formalisation of joint-working arrangements were highlighted as a way to address the current challenges in the specialism.

**Conclusions:**

Findings demonstrate how informal and formal relationships between organisations and individuals, enabled early communication with families, and collaborative working with specialist services. Formalising these could increase knowledge and awareness of end of life care, improve staff confidence, and overall improve professionals’ experiences of delivering care, and families’ experiences of receiving it.

There are considerable positives that come from collaborative working between different organisations and sectors, and care could be improved if these approaches are funded and formalised. There needs to be consistent funding for paediatric palliative care and there is a clear need for education and training to improve staff knowledge and confidence.

**Supplementary Information:**

The online version contains supplementary material available at 10.1186/s12904-023-01238-w.

## Background

In the UK, paediatric end-of-life care has changed considerably in the last 30 years [1, 2]. Additional children’s hospices have been established, specialist training in paediatric palliative medicine and nursing has been developed [2], and there has been an increase in the number of children requiring this type of care [3]. Nevertheless, evidence on paediatric end-of-life care in the UK has consistently shown that its provision and utilisation is inequitable [4, 5]. Reasons for this include the unequal geographical distribution of key services [5], with paediatric end-of-life care being described as “patchy” [6]; and because care is often coordinated across a number of different organisations and sectors, each with their own funding sources and models of care [7, 8]. This inequity in delivery and access, contributes towards poorer outcomes for many families. For example, in instances where out-of-hours support is “sparse or non-existent”, children and their families have a reduced choice over place of care [9].

There have been considerable efforts to address these inequities, although this has largely not been supported with statutory funding [1, 10], or by a national-level strategy, until the introduction of NHS England’s PEoLC (palliative and end of life care) Strategic Clinical Networks [11]. Prior to this, initiatives included the publication of guidance for the provision of end-of-life care for children [12], and the development of informal regional children’s Palliative Care Networks (PCNs) to coordinate care [13, 14]. Whilst these networks work in different ways, their aim is to bring together the professionals and organisations responsible for children’s end-of-life care in each region, across the UK [14]. There is also now a move to establish more formally managed clinical networks to further enhance the planning and delivery of joined up care. Nevertheless, the inequity in the provision of, and access to end-of-life care for children still remains [5, 13].

Evidence on the reasons for inequities in paediatric end-of-life care, both inside and outside of the UK, tends to be limited to the experiences of those working in a specific context [15], or with children with certain conditions [16], and/or focused on specific elements of end-of-life care, such as symptom management [1, 17]. Consequently, it is difficult to gain an overarching understanding of the recent successes, and continuing challenges to attempts to improve end-of-life care in the UK. The Chairs of the UK’s regional PCNs are in a unique position to provide this understanding.

This study aimed to explore the views of the Chairs of the regional PCNs of the successes of, and challenges to the equitable coordination and delivery of end-of-life care for children in the UK.

## Methods

This study forms part of a wider research programme on end-of-life care for children in the UK, the ENHANCE study [18]. As part of the first workstream, we conducted a qualitative study grounded in an interpretivist approach, using semi-structured interviews with the Chairs of the regional PCNs from across the UK.

### Sample and recruitment

At the time of data collection, there were 16 PCNs across the UK [14]. In some instances, these networks are now Managed Clinical Networks (MCNs) [13].

Contact details for the PCN Chairs were obtained from the Together for Short Lives directory [14]. Participants were invited to participate in the study via email, which included a study flyer and a participant information sheet. If no initial response was received, up to three follow-up emails were sent over a period of six weeks. Participation was discussed further by email, and written consent was obtained electronically prior to participation.

### Data collection

Semi-structured interviews were conducted via video call by authors AP, JH and BB (one male and two female, applied health researchers, previously unknown to participants). Data were collected between October-December 2021. Topic guides (see Supplementary file) were developed for this study with reference to the relevant literature and in relation to the content of surveys that were conducted as part of the ENHANCE study [18].

### Data analysis

All interviews were audio-recorded and transcribed verbatim. Data were analysed thematically using a coding process that was initially informed by the study aims and the topic guides [19]. First, one author (AP) read and re-read the transcripts to facilitate data immersion. AP then conducted the initial coding process using eclectic coding techniques, which included in-vivo codes and the “lump” coding of meaningful segments of the data that were later split into multiple smaller codes [20]. These initial codes were grouped into descriptive categories through axial coding [21], which entailed bringing together the connections between codes. The descriptive categories were then organised into preliminary analytical themes through selective coding [21]. Themes were developed and refined iteratively with JH and subsequent discussion with the team. NVivo (Version 12) was used to support this process.

### Patient and public involvement

For this study, there was a Parent Advisory Panel (PAP), consisting of around 13 bereaved parents, who advised us at all stages of the research. For example: they reviewed the study proposal and actively contributed to the study design. They reviewed all study documentation and refined the interview topic guide. They provided feedback on initial analytical themes, providing new avenues of thoughts, and clarified aspects of the data.

## Results

We conducted 16 interviews with 19 participants, representing 15 of the 16 PCNs, across all areas of the UK. At the time of data collection, some of the PCNs had joint chairs; in some instances, we interviewed two co-chairs together in one interview. The average interview duration was 47 min (range: 35–63 min). The inter-related analytical themes developed are detailed below and in Table 1.

**Table 1:** Description of themes identified

**Standalone theme 1:** Communication during end-of-life care *Care is affected by how and when health professionals communicate about end-of-life decisions with children and their families*		
**Overarching theme 2:** Getting end-of-life services and staff in the right place *The coordination and delivery of paediatric end-of-life care can be inequitable because of challenges around services and staffing*		
**Theme**	**Description of theme**	**Sub-themes**
1. Access to, and staffing of end-of-life care	Paediatric end-of-life care is not always equitable because of the nature of palliative care, the way that services were developed historically, and because of staff shortages	a. Access to end-of-life care services b. Large areas, small numbers, high intensity c. Staff shortages and “goodwill” d. 24/7 advice lines and regional networks
2. Inconsistent and insufficient funding for end-of-life care services	Funding for paediatric end-of-life care services can be difficult to obtain. When it is available, it can be unreliable	a. Difficulties sourcing and maintaining sufficient funding b. Reliance on charitable organisations
**Overarching theme 3:** Linking up healthcare provision *The challenges and successes that come from the requirement for end-of-life care to be coordinated and delivered across different services, organisations and locations*		
**Theme**	**Description of theme**	
1. Coordination successes	Positives that result from formal and informal relationships between services	
2. Role of the networks	Networks would be best placed to reduce equity in end-of-life care	
3. Coordination challenges	Difficulties caused by having to work across organisational and administrative boundaries	

### Standalone theme 1: communication with families about end-of-life care

Network Chairs described how the successful coordination and delivery of end-of-life care could be dependent on communication between professionals and children and their families.

*“I feel quite passionately that a lot of children die in intensive care because we’re not good at having those conversations.” *Participant 5

Many of the Network Chairs believed this was particularly an issue when conversations about end-of-life care took place late in a child’s treatment as this reduced the amount of time there was to organise care. Some Network Chairs explained this was often the case for children with diagnoses that meant they followed a curative pathway, or for those children whose prognosis changed quickly or unexpectedly.

*“Sometimes with the oncology children or the cardiac children, the curative focus means there isn’t the recognition that actually that child is now palliative and they die quite suddenly in an acute placement, which actually may not be the preferred choice of place of death.” *Participant 2

*“I think that for neurodisability […] it can be about recognition about when it’s appropriate to involve a hospice service, for example. Because this can be unclear for this population.” *Participant 9

In contrast, when these conversations could happen in a timely manner, initiatives like advance care planning could take place, which Network Chairs identified as helping services provide the right care at the right time.

*“If we can do something positive, it’s changing that culture slightly, so we have those conversations about what parents would want, and [talk about] what point […] you switch to a purely palliative model.” *Participant 5

Several positive experiences that had resulted from good, early communication were highlighted, including instances when children and their families were empowered to make informed choices.

*“The parents spoke to the clinicians very frequently [and] they talked through the options. When he died he was pain-free, very comfortable, in the arms of his mummy and daddy, so that is a tick and [a] star, I think, for everybody that was involved. It was very good.” *Participant 11

*“Actually the parents were just fearful. What they were used to was hospital care because this little boy had had treatment in hospital for most of his life […] By empowering them, by giving them the right support, they chose to stay at home and not go to hospital.”* Participant 2

One Network Chair felt that parents’ engagement with paediatric end-of-life care could be driven by the language that health professionals use, and this could also be a contributor towards ethnic and socio-cultural inequities in the coordination and delivery of care.

*“We’re exploring whether to use the term hospice because a lot of families, particularly in certain cultures, don’t like the word. For some, English isn’t their first language, they don’t like the word hospice […] or they haven’t heard of us.” *Participant 10

### Overarching theme 2: getting end-of-life services and staff in the right place

This overarching theme explains how the coordination and delivery of paediatric end-of-life care can be inequitable because of challenges around services and staffing.

### Theme 1: access to, and staffing of end-of-life care

The Network Chairs believed end-of-life care was made difficult by the way services have developed historically, by the nature of paediatric end-of-life care itself, and because of staffing shortages.

#### Sub-theme a: access to end-of-life care services

Most of the Network Chairs believed that the location of key services could influence some of the inequity in end-of-life care outcomes and experiences. The challenges were often different in urban areas versus rural areas. In the former, some Network Chairs said families generally had more choice about place of care because there were several services available to them, and this meant health professionals with end-of-life care experience had to support more services. In the latter, some Network Chairs described health professionals as being more stretched, and it being difficult for families to access services, often because of travel times.

*“In a large city it would be easier to cobble together a 24-hour rota, because there are more nurses around and therefore it’s more easily sustainable. Whereas in rural areas, it would be much harder to do that.” *Participant 1

*“If you have children who live a long way from the hospice, it’s really hard to get end-of-life hospice care at home because the staff are unwilling to travel two hours to do a shift and then two hours back.” *Participant 16

These challenges were very dependent on the local context, and the differences between urban and rural areas were not always intuitive.

*“From my experience, families who live in a more rural area the local community are much more prepared to support them than families that are in an urban area.” *Participant 7

*“Being close to specialist services is the problem. The orientation of many of the Trusts towards a hub beyond the region complicates life for us. *Participant 9

The Network chairs also described how issues of access could interact with the socio-economic status or cultural backgrounds of families.

*“If they’re from the other side of the patch and they’re from a low socioeconomic group or they culturally don’t want to be displaced from the rest of their family, those children get a poor service.” *Participant 13

#### Sub-theme b: large areas, small numbers, high intensity

Many of the Network Chairs said the provision of specialist/intensive care packages required the input of experienced and well-trained staff. However, this care was usually only required by a very small number of children at any one time, with each child requiring a tailored approach.

*“There isn’t one size that works every time, it is very much taking each case as it comes along […] and finding a novel solution, which everyone recognises is not sustainable and not ideal.” *Participant 2

This could mean some children received different care than others, particularly if they were cared for by staff who had less experience of palliative care.

Because not all areas had the same services or staff expertise available for families to access, care often had to be organised ad-hoc on each occasion it was required. This was especially true in regions that were more rural where the children were usually spread over a large geographical area.

*“The problem is that we are dealing with small numbers, but high intensity when they happen and a very widespread geography in our region.” *Participant 3

Some Network Chairs believed the lack of defined standards in the sector meant it was difficult to guarantee equity between different service patches.

“*If I had a child in this hospice, would they get the same in the next hospice? There is no expectation of what a hospice actually delivers and what are the core principles.” *Participant 12

#### Sub-theme c: staff shortages and “goodwill”

Most Network Chairs said there was a shortage of staff to work in paediatric end-of-life care. Some regions had had difficulties when recruiting to posts that required a particular level of skill and/or experience, and had to redefine these posts to be able to appoint staff to them. Equally, some of the Network Chairs said it had become clear how reliant some services and regions were on the skills, experience and vision of a few individuals who had now left the region or retired.

Issues like these have meant many staff who end up delivering end-of-life care were perceived by the Network Chairs as inexperienced or lacking confidence when supporting these children and their families.

*“I think the consultant who knew the child previously wasn’t happy to take the child over for end-of-life care […] She felt out of her depth.” *Participant 16

*“I’m aware of a number of really challenging cases where staff have been put in really difficult positions because they haven't had the medical support that they needed for end-of-life care […] That’s nobody’s fault, it’s just that there is not the expertise.” *Participant 15

Because so much end-of-life care was being provided by health professionals who may be less experienced or lacking confidence, many Network Chairs believed this had contributed to less effective communication with children and their families.

In some cases, the lack of experience and confidence was felt to have been due to the small number of children requiring paediatric end-of-life care, because these children may not be the focus of health professionals’ time and skills.

*“A nurse said to me: “On the ward if you had five children and one was receiving end-of-life care, they were almost your least priority because you had four other children who you were giving acute care to,” and that broke my heart.” *Participant 10

The small number of children also made it difficult to justify, and demonstrate, the need for certain services or skills to work in this specialism.

*“They’re one-off things that perhaps you can’t write, “There’s a need for X number of young people to have this service available to them,” because it’s often a one-off thing.” *Participant 11

*“If you look at that hard data it is infrequent that the doctor who is on call actually gets a call out of hours. Why on earth would you need a level four consultant available twenty-four hours a day, seven days a week?” *Participant 6

According to the Network Chairs, the ability to be flexible in terms of resource use, and the training and safety of staff were affected by the lack of standards in the sector.

*“Do you have one nurse on, do you have two nurses on? That type of stuff. Is it safe in a rough area for one nurse overnight to walk in or do you have police escort? There’s all those sorts of issues as well, so there’s no clear guidance.” *Participant 12

These challenges, as well as the complexity, intensity and unpredictability of paediatric end-of-life care, meant a large number of the hours of end-of-life care provided, and the administrative work in coordinating that effort, came from the “goodwill” of individual health professionals.

*“Care during the end-of-life period still relies very heavily on individual goodwill and nurses don’t get paid for it. And if it goes on for more than a couple of weeks, they very quickly get exhausted.” *Participant 1

*“We don’t just go, “There’s nobody to do it, oh well let’s go home,” we’ll actually try and do something about it to sort it for the family and for the young person. *Participant 11

#### Sub-theme d: 24/7 advice lines and regional networks

Most of the Network Chairs were seeking to establish 24/7 telephone advice lines to help address this mismatch between staffing expertise and the needs of children. They believed that these could allow professionals to consult with other colleagues who are more skilled or experienced, thus reducing some of the barriers caused by needing to provide intensive treatment over a large geographical area.

*“Our telephone advice line would be for the children’s community nurses who would be working 24/7 […] That way we can keep the children at home and they don’t have to bounce back into hospital in the night if they become unstable.” *Participant 13

The Chairs also highlighted the role of the palliative care networks in addressing issues with staffing.

*“The Network’s there for support of staff, and that may be through education […] We have the clinical subgroups, so we can have meetings where individual consultants and nurses can get peer support.” *Participant 13

One Network Chair believed many of the issues outlined in this theme could be solved through the development of regional-based strategies; in their region, a similar initiative had provided clear targets and a rationale for commissioners.

### Theme 2: lack of consistent and sufficient funding for end-of-life care services

Funding was mentioned by all Network Chairs as being an important issue affecting paediatric end-of-life care services.

#### Sub-theme a: difficulties sourcing and maintaining sufficient funding

The majority of the Network Chairs talked about how difficult it was, and how long it could take, to obtain consistent funding for the services they believe their region needs.

*“We then had to go and meet with the Trustees of the Trust to talk about why they should prioritise us. We were competing with all the big adult services, all the big transplant stuff, all the big oncology stuff, everything.” *Participant 11

*“Two years ago, we put together a bid to the CCGs to get some funding for a paediatric palliative care consultant. The money I believe, having tried to chase it […] is just about there. *Participant 14

Network Chairs said their ability to advocate for more resources for the specialism at a national level was often related to two issues: firstly, the fact that children’s end-of-life care has a low national profile, largely because of the small numbers of children; and secondly, the lack of outcome measures in paediatric end-of-life care made it difficult to demonstrate benefits to funders.

*“These children are a very small part of the agenda. [We need to] make sure that children aren’t forgotten in terms of national policymaking and their needs are addressed. The small numbers are also a challenge […] to our ability to deliver a workforce.” *Participant 9

*“We’re not clear as a sector to funding bodies about what is fundable and why we would want it funded for a particular level […] If we can’t define ourselves well and clearly, and be honest about that, then I think we’re kind of sabotaging ourselves.” *Participant 12

Network Chairs said this difficulty of securing consistent funding also applied to the regional networks, with the work to source funding for key roles within the networks, such as the network coordinators, sometimes getting in the way of efforts to provide front-line services.

#### Sub-theme b: reliance on charitable organisations

Many Network Chairs were keen to highlight that many important services and key posts in their region were provided by the charitable sector and receive very little NHS funding.

*“The local hospice, in effect, provides the end-of-life community nursing service for the region rather than the NHS.” *Participant 15

*“The first consultant, who has been in post now four years, was funded by a charity […] These are pivotal posts. They shouldn’t necessarily be reliant on charity funding to start off really.” *Participant 8

One Network Chair talked about the reliance on hospice organisations, saying that hospices were unable to care for children if they had filled their beds or did not have staffing capacity. This could affect the equity of care provided to children through a narrowing of options available to parents.

### Overarching theme 3: linking up healthcare provision

This overarching theme explains how Network Chairs talked about the successes and challenges of when services work together to provide paediatric end-of-life care in their region.

### Theme 1: coordination successes

Most Network Chairs talked about the positives that resulted from formal and informal relationships between services. These were facilitated through: regular meetings between providers working jointly; secondments or link people working across more than one service, which can increase referrals to end-of-life care providers; and good, regular communication between health professionals.

*“We have a monthly palliative care meeting where cases can be brought and that’s a real MDT (multi-disciplinary team) kind of perspective and we can make the necessary referrals easily.” *Participant 7

*“I’ve got someone in my team who also works for a cancer service and my trainee is based at the hospital. Them being there, boots on the ground, are more likely to spot people that need a referral.” *Participant 4

*“It is down to good communication […] it’s building the relationships and it relies on those partners ensuring everybody is informed and up to date. *Participant 2

The Network Chairs said that because of the small size of the specialism, many of the practitioners also had good personal relationships with those who worked in other areas and regions, and their informal communication helped to facilitate care across different services.

### Theme 2: role of the networks

For most of the regions, the Networks had facilitated the informal relationships between health practitioners and the sharing of best practice.

*“I think the network helps, having those relationships, knowing each other, knowing what’s possible.” *Participant 9

*“The network is a really good way of sharing what we’re doing, and I think it probably enhances peoples’ confidence to pick up the phone to different people because they’ve seen them at the network meetings.” *Participant 7

This varied by region, however, with some Network Chairs saying that their region did not have a formal structure or governance process in place, with care generally being provided by Trusts or Health Boards on an individual basis. Many believed that future work to reduce equity in end-of-life care would be best facilitated through the Networks.

### Theme 3: coordination challenges

Several of the Network Chairs highlighted the difficulties caused by having to work across organisational and administrative boundaries, describing service provision as often being confusing to navigate.

*“One of those children who died was discharged to our service and they had five different appointments in the next eight weeks with different people, or different tests back in the city centre; this for a child that lived 50 miles away.” *Participant 14

The lack of a common system for data sharing was a major part of this difficulty and was more notable in some regions than others.

*“Whenever a child crosses a boundary […] moves from a hospital to a community service or to a hospice, then the information has got to be transferred. You can find that you’re visiting a child at home with the community nurse and there’s no record.” *Participant 3

Funding agreements could also get in the way of providing a more joined-up service.

*“We tried to amalgamate two teams, but it just became a really challenging thing to do because of ownership of funding. We just couldn’t blend those two services together.” *Participant 8

Some Network Chairs had very different experiences of this challenge. For example, in one region the issue was that some key services were located in other regions, whereas for another region, the issue was that the patch was so large that it was difficult to share staff between services.

## Discussion

### Summary

This study provides an overarching viewpoint on the coordination and delivery of end-of-life care for children in the UK, from the perspectives of Network Chairs of the PCNs. It has highlighted some of the successes of those working in end-of-life care and presented potential solutions for the challenges currently faced in this specialism.

Findings show that good communication between health professionals and children and their families is very important for end-of-life care to be successful. Findings also highlight difficulties in organising staff and services for children at the end of their lives, including the current staffing shortages and the inconsistent funding arrangements that exist for this type of care. The extent and impact of these challenges varied between and within regions [22]. There have also been significant successes resulting from formal and informal relationships between organisations and individuals, which Network Chairs believe has led to improved communication between services and increased referrals for end-of-life care.

### Contribution to the wider literature

Contrary to much of the research in this area [1, 10, 13, 23], the Network Chairs gave several examples of when paediatric end-of-life care had gone well. These successes were often due to good, early communication with children and/or their families. This supports previous research that has suggested those who receive the best end-of-life care are those whom are well informed and receive timely advice, and that trusting relationships are valued by families [1, 23, 24]. Most of the Network Chairs mentioned Advance Care Plans as being a good tool to facilitate good communication, but some existing evidence suggests they are not always acceptable to families [25], so we suggest that care should be taken to ensure flexibility in their introduction, timing and management.

Current literature suggests a number of reasons why good communication cannot always be achieved, such as the speed of progression of a child’s condition, or because the child initially received care on a curative pathway, both of which may make it difficult to recognise the most appropriate moment to have end-of-life conversations [25, 26]. Another reason is the cultural ‘collusion of immortality’ that exists in the healthcare system, whereby there is widespread denial of the possibility of dying in the hope of ‘miracle recoveries’, which is further exacerbated by the way health systems are organised into specialisms with no one specialism taking holistic responsibility for a patient’s care [1]. This study has highlighted that the current staff shortage [27] may also be an important issue as its implications for paediatric end-of-life care include reduced staff expertise, experience and confidence, all of which can have an impact on communication [25, 26, 28–31]. This finding, and previous research [32, 33], suggests it is important for the goals of end-of-life care to be made clear to all health professionals [34], and staff training and education about end-of-life care across the health sectors is likely to have a positive impact on care delivery and coordination [13, 35, 36].

In terms of solutions, the presence of ‘role models’, such as specialist palliative care team members, have been shown in other research to have a positive impact in boosting awareness of end-of-life issues amongst all health professionals [28]. The Network Chairs believed 24/7 advice lines could also help support health professionals to provide end-of-life care. The informal and formal relationships between organisations and individuals highlighted in this study appeared to be providing similar benefits and could be encouraged and formalised. The Network Chairs believed these relationships also helped to increase the awareness of end-of-life care amongst health professionals generally and increased referrals to end-of-life care services. Increasing referrals to specialist services or services with greater experience of paediatric end-of-life care could reduce the chances of end-of-life conversations taking place late in a child’s care [37], as well as increasing the inclusivity of care, particularly with respect to ethnicity and socio-economic status [38, 39].

Other factors highlighted by the Network Chairs in this study as having the potential to negatively affect paediatric end-of-life care included the location and availability of services, service coverage and organisation, and funding. All of these challenges were closely related to other factors such as staffing and to the differences between, for example, cancer and non-cancer services [13, 34, 23, 2, 10].

This study showed that within-region variation in service location, funding and staffing (among other factors) was an important driver of the geographical disparity in end-of-life care services for children. Travel times and the complexity and intensity of care have been highlighted in other studies as being particular challenges for providing palliative care in rural regions [40, 41]. However, this has tended to be presented as a straightforward rural–urban divide, whereas some of the Network Chairs in this study believed that this relationship was more complicated and this suggests a more nuanced view of these challenges is required. This study was also able to illustrate tangible impacts of staff and funding shortages, highlighting the large amount of care being provided through the “goodwill” of health professionals. The reliance on non-statutory funding for important services was also an important concern for Network Chairs.

Finally, this study has shown that the coordination and delivery of palliative care is complex and multifaceted [40]. For example, the inability of service leaders to justify certain services and obtain consistent funding can be said to be partly due to the low profile of the sector because of the small number of children requiring end-of-life care. It is, therefore, encouraging that the new amendment to the Health and Care Bill now makes it a legal duty for Integrated Care Boards in England to commission palliative care for people of all ages [42]. There will be a need to recognise that end-of-life care for children is different from adult end-of-life care [12]. The Network Chairs believed the lack of agreed-upon standards and outcome measures for paediatric end-of-life care also affected the training and development of staff and the demonstration of the benefits of end-of-life care to commissioners. The research currently underway to develop new outcomes and experiences measures [43–45], and the work to ensure all regions meet the NICE guidance, will go some way towards alleviating this. This study has noted the extent to which the identified challenges varied by region, so it is likely that some regional-specific initiatives will be required to fully address the current inequity in the delivery and coordination of end-of-life care for children in the UK. This would in turn, go some way to reaching equal outcomes for children, young people, and their families.

### Study strengths and limitations

The findings of the study are based on the views of 19 Network Chairs and provide a unique perspective and overview on end-of-life care. It is probable that other health professionals who work in the same regions as the Network Chairs will have different views [13], but it is a strength of the study that it has elicited the opinions of those individuals who have been actively involved in addressing some of the challenges they have highlighted. This study has also provided important context for the analysis of other data collected for a wider programme of work [18]. Within which, the perspectives of parents who have received end-of-life care for their infant, child, or young person; and the professionals who have delivered end-of-life care for these groups, will be explored.

## Conclusions

This study has demonstrated that whilst the coordination and delivery of palliative care is complex and multifaceted, the informal and formal relationships between organisations and individuals, clearly enabled early communication with families, and collaborative working with specialist services, and should be encouraged and formalised. This could go some way to increasing knowledge and awareness of end-of-life care, improving staff confidence, and therefore increasing referrals to specialist services. In turn, this would reduce the chances of end-of-life conversations taking place late in a child’s care, improving delivery and experiences of end-of-life care, and therefore outcomes. The challenges faced, vary by region and so initiatives need to be sensitive to different local contexts.

### Supplementary Information

**Additional file 1.** Interviews with leads/chairs of palliative care networks.

## Data Availability

Data are available from the corresponding author upon reasonable request.
