# Supplementary material for: Regional perspectives on the coordination and delivery of paediatric end-of-life care in the UK: a qualitative study
Source: BMC Palliat Care. 2023 Aug 16;22:117. doi: 10.1186/s12904-023-01238-w (PMC10428585; doi:10.1186/s12904-023-01238-w)
Supplement: Supplementary file 1 — Additional file 1. Interviews with leads/chairs of palliative care networks. [file 12904_2023_1238_MOESM1_ESM.docx]

**Interviews with leads/chairs of palliative care networks**

**INTRODUCTION**

- Thank for time
- Confirm consent
- Brief reminder of purpose of the study and role and focus of interview

**MAPPING END OF LIFE/PALLIATIVE CARE SERVICES IN THE REGION**

1. Neo-natal, paediatric and young adult palliative care services in the region:
   - 1. Type(s) of care service provides
     2. Acute and/or community
     3. Funding/commissioning
     4. Location and geographical reach
     5. Population
2. Professionals posts and specialist training:
   1. Location
   2. Role and population
   3. Funding arrangements
3. Equity of access to end-of-life care / palliative care services:
4. geography/place
5. age
6. diagnosis
7. Examples of provision / care pathways within region.

**IMPROVING / DEVELOPING END OF LIFE / PALLIATIVE CARE FOR BABIES, CHILDREN AND YOUNG PEOPLE IN THE REGION**

1. Needs in region for babies, children and young people who are at end of life.
2. Barriers and facilitators.

**FACTORS THAT MOST AFFECT OUTCOMES AND EXPERIENCES AT END OF LIFE**

1. Needs in *acute and community setting*s to ensure good and holistic end-of-life care of babies, children and young people.

**THE REGIONAL NETWORKS**

1. Explore:
   1. Views purpose of networks
   2. Views on extent to which this is being achieved in region
   3. Views on barriers / what’s needed.

**CLOSE**
